# Bioactive Phospholipids Enhance Migration and Adhesion of Human Leukemic Cells by Inhibiting Heme Oxygenase 1 (HO-1) and Inducible Nitric Oxygenase Synthase (iNOS) in a p38 MAPK-Dependent Manner

**DOI:** 10.1007/s12015-018-9853-6

**Published:** 2018-10-09

**Authors:** Ahmed Abdelbaset-Ismail, Monika Cymer, Sylwia Borkowska-Rzeszotek, Katarzyna Brzeźniakiewicz-Janus, Pranela Rameshwar, Sham S. Kakar, Janina Ratajczak, Mariusz Z. Ratajczak

**Affiliations:** 1 Stem Cell Institute at James Graham Brown Cancer Center, University of Louisville, 500 S. Floyd Street, Rm. 107, Louisville, KY 40202 USA; 2 Faculty of Veterinary Medicine, Zagazig University, Zagazig, Egypt; 3 Center for Preclinical Research and Technology, Department of Regenerative Medicine, Warsaw Medical University, Warsaw, Poland; 4 Department of Physiology, Pomeranian Medical University, Szczecin, Poland; 5 Department of Hematology, Multi-specialist Hospital, University of Zielona Gora, Gorzow Wlkp, Poland; 6 Rutgers New Jersey Medical School, Newark, NJ USA

**Keywords:** Leukemia, S1P, C1P, LPA, LPC, HO-1, p38 MAPK, HO-1 activators

## Abstract

Bioactive phospholipids, including sphingosine-1-phosphate (S1P), ceramide-1-phosphate (C1P), lysophosphatidylcholine (LPC), and its derivative lysophosphatidic acid (LPA), have emerged as important mediators regulating the trafficking of normal and cancer cells. While the role of S1P in regulating migration of hematopoietic cells is well established, in this work we compared its biological effects to the effects of C1P, LPC, and LPA. We employed 10 human myeloid and lymphoid cell lines as well as blasts from AML patients. We observed that human leukemic cells express functional receptors for phospholipids and respond to stimulation by phosphorylation of p42/44 MAPK and AKT. We also found that bioactive phospholipids enhanced cell migration and adhesion of leukemic cells by downregulating expression of HO-1 and iNOS in a p38 MAPK-dependent manner but did not affect cell proliferation. By contrast, downregulation of p38 MAPK by SB203580 enhanced expression of HO-1 and iNOS and decreased migration of leukemic cells in vitro and their seeding efficiency to vital organs in vivo after injection into immunodeficient mice. Based on these findings, we demonstrate that, besides S1P, human leukemic cells also respond to C1P, LPC, and LPA. Since the prometastatic effects of bioactive phospholipids in vivo were mediated, at least in part, by downregulating HO-1 and iNOS expression in a p38 MAPK-dependent manner, we propose that inhibitors of p38 MAPK or stimulators of HO-1 activity will find application in inhibiting the spread of leukemic cells in response to bioactive phospholipids.

## Introduction

Evidence has accumulated that, in addition to well-known peptide-based factors, including growth factors, cytokines, and chemokines, bioactive phospholipids also modulate the migration of normal and malignant cells [1–7]. Importantly, these lipid-based molecules are already present at biologically relevant concentrations in tissues and blood plasma, and their levels increase in several situations related to organ/tissue damage. We have recently proposed that these pro-migratory factors increase in the body after radio-chemotherapy, which may promote the unwanted spread of resistant malignant cells that have survived antileukemic treatment [2, 8].

Here we focus on the biological effects of phospholipids, including ceramide-1-phosphate (C1P), lysophosphatidylcholine (LPC), and its derivative lysophosphatidic acid (LPA), on malignant human hematopoietic cells. We compared the effects of these phospholipids with the best-studied member of this family, S1P, and with the chemokine stromal-derived factor 1 (SDF-1). The first two phospholipids, S1P and C1P, belong to the family of phosphosphingolipids [5, 7, 9]. The two others, LPC and LPA, are phospholipids, and LPA is a product of enzymatic modification of LPC by the enzyme autotaxin [10, 11]. With the exception of C1P, the receptors for these phospholipids have been cloned and found to be expressed on the surface of several types of normal and malignant cells.

The rationale for performing this study was that, in contrast to S1P, the effects of C1P, LPC, and LPA on leukemic cells are still not well known. Specifically, while S1P has been reported to be involved in the pathogenesis of CML, AML, ALL, and multiple myeloma and to chemoattract leukemic cell lines [12–15], the effects of a second bioactive phosphosphingolipid, C1P, on leukemic cells (except its effect on the migration of murine RAW264.7 macrophages) [16] have so far been understudied. Similarly, there is very limited information about the effects of LPC and LPA on leukemic cells.

Based on the biological effects of S1P on leukemic cells, small-molecule inhibitors of enzymes involved in S1P synthesis, e.g., sphingosine kinase 1 and sphingosine kinase 2, have been proposed for treatment of patients [17–22]. However, one has to remember that bioactive lipids are present in the tissues and body fluids as a mixture of different molecules and that simply inhibiting one bioactive phospholipid–receptor axis (e.g., S1P–S1P type 1 receptor) may not be sufficient, as other compounds may compensate for this inhibition by stimulating leukemic cells on their own.

While considering the development of bioactive lipid inhibitors, one has to recognize that these molecules signal through several cell-surface receptors [4, 23]. For example, S1P interacts with five different receptors (S1PR_1–5_) [1, 2, 4, 23], LPA activates five receptors (LPAR_1–5_) [24–26], and LPC activates G2A and GPR4 [27, 28]. All these are G protein-coupled receptors. Therefore, strategies to inhibit leukemic cell motility by blocking one of the receptors would be ineffective [29–34], and thus targeting common signaling molecules located downstream of these cell-surface receptors would be more effective. Our recent work on normal hematopoietic cells as well as solid cancer cell lines revealed that cell migration can be efficiently inhibited by upregulating the intracellular activity of heme oxygenase 1 (HO-1) [35–38] or inducible nitric oxide synthetase (iNOS) [39].

In the work reported here we found that bioactive phospholipids enhanced cell migration and adhesion of leukemic cells by downregulating expression of HO-1 and iNOS in a p38 MAPK-dependent manner but did not affect cell proliferation. Based on these findings, inhibitors of p38 MAPK may find application in inhibiting the spread of therapy-resistant leukemic cells in response to S1P, C1P, LPC, and LPA gradients.

## Materials and Methods

### Human Hematopoietic Cell Lines

Ten human malignant hematopoietic cell lines, including seven myeloid (HEL, K-562, U937, KG-1a, HL-60, DAMI, and THP-1) and three lymphoid (NALM-6, JURKAT, and DAUDI) cell lines, were used in this study (and obtained from the ATCC). All these cell lines were cultured in Roswell Park Memorial Institute (RPMI) medium 1640 containing L-glutamine (GE Healthcare) and 10% heat-inactivated fetal bovine serum (FBS; Seradigm), 100 units/mL penicillin, and 10 μg/mL streptomycin (Corning) and incubated in a humidified atmosphere of 5% CO_2_ at 37 °C, with exchange of medium every 48 h.

### Acute Myeloid Leukemia (AML) Patients

Patients with newly diagnosed AML (*n* = 7) were employed in this study. Such a diagnosis was made according to the WHO classification system [40]. Determination of complete blood counts and flow cytometry were performed to confirm the existence of blast cells. EDTA-anticoagulated whole blood was collected from these AML patients, and the peripheral blood mononuclear cells (PB-MNCs) were immediately separated by density-gradient centrifugation using Histopaque 1077 medium (Sigma-Aldrich). Patients samples were obtained for this study according to Institutional IRB guidelines.

### CD33^+^ AML Blasts

CD33^+^ blasts were sorted from AML patient samples using magnetic cell sorting (MACS, Miltenyi Biotec), according to the manufacturer’s instructions. In brief, the PB-MNCs obtained were labeled with MicroBeads conjugated to mouse anti-human anti-CD33 antibodies (Militenyi Biotec) by incubation in staining buffer (autoMACS running buffer) for 30 min in 4 °C. CD33^+^ blasts were harvested from the column after the magnetic field was switched off.

### Reverse Transcriptase-Polymerase Chain Reaction (RT-PCR)

mRNA was extracted and purified from cells using the RNeasy Mini kit (Qiagen Inc.) after DNase I (Qiagen Inc.) treatment. The purified mRNA was reverse-transcribed into cDNA using Taqman Reverse Transcription reagents (Applied Biosystems), and the synthesized cDNA fragments were amplified using Amplitaq Gold polymerase (Applied Biosystems). The human sequence-specific primers employed for amplification are shown in Table 1. The PCR conditions were: 1 cycle of 8 min at 95 °C; 2 cycles of 2 min at 95 °C, 1 min at 60 °C, and 1 min at 72 °C; 40 cycles of 30 s at 95 °C, 1 min at 60 °C, and 1 min at 72 °C; and 1 cycle of 10 min at 72 °C. Samples without template controls and reverse transcriptase were used in each run. All primers were designed using the NCBI Primer-BLAST program, and at least one primer included an exon–intron boundary. Afterwards, all PCR products were analyzed by 2% agarose gel electrophoresis.

**Table 1:** Sequences of primers employed in PCR studies

Name	Forward primer sequence	Reverse primer sequence
S1PR_1_	GAACAGCATTAAACTGACCTCGG	CAAACATACTCCCTTCCCGC
S1PR_2_	CCGATTTCTCCTTTTCGAATGT	AAATAGTGCAACTGAGCAATGAGG
S1PR_3_	GCAGCACTTCAGAATGGGATC	GGCAGTGGATGATGTCAGCA
S1PR_4_	TTCTGACGCCAAATGGGC	GATCGAACTTCAATGTTGCCAG
S1PR_5_	CCCCACAATGTGAACAAACAGA	TCCCTCATCCTGAAATGCTTTT
LPAR_1_	GCAGCTCCACACACGGATG	TAGTCCTCTGGCGAACATAGCC
LPAR_2_	CTGTCGAGCCTGCTTGTCTTC	CAGCCTAAACCATCCAGGAGC
LPAR_3_	TGTCTCCGCATACAAGTGGG	GGGTTCACGACGGAGTTGAG
LPAR_4_	TACAACTTCAACCGCCACTGG	ACATTAGTGGTGGAAAACAAAGAGG
LPAR_5_	GATGAAGCTGTGACCAAACGC	CATGGTCCCAAAACAAGCAGA
G2A	GAGCGTCTGTCAGCGGAGTC	CGTGTGCCAGGATTTCCCT
GPR4	CCAGTTTTCCCCTCTCATCC	CACAGCCCTTTTTCCATACAA
SK1	TGAGCAGGTCACCAATGAAG	GGCTGAGCACAGAGAAGAGG
SK2	ACAACGAGGAGAGCTGCAAT	AGAAGTCCAGGCTGGTGAGA
SPP1	GCCGCTCTACTGCCTGTT	ATGACCAGCACCCAGATGA
SPP2	ATGCATACGGTCCTGGATGT	TATGACACACACGGGGAAGA
S1p lyase	ATACTGATGGCCTGCAAAGC	TCCCAAAGTAACTGGCTGCT
CERK	GAGAAGCTGACGTCCAGACC	CCTTGGCCTGATTAGCATGT
LPP1	AGGGAGCTCTGGTTGCAATA	TCCCAGTTGTTGGTGTTTCA
LPP2	TACACCCGCGTGTCTGATTA	TCCTTCAGACAGTGCTGTGG
LPP3	CCTCATCATCGAGACAAGCA	CACAGAGCACAGCGTCATTT
autotaxin	AAGGCAAAGAGAACACGCTG	ATCTGACACGACTGGAACGAG
β-actin	GGATGCAGAAGGAGATCACTG	CGATCCACACGGAGTACTTG

### Quantitative Real-Time PCR (RT-qPCR)

After pretreatment with or without SB203580, a p38 MAPK inhibitor (20 μmol/L) (Selleckchem, USA), the cells were stimulated in vitro with S1P, C1P, LPA, LPC, or SDF-1. The purified RNA was reverse-transcribed with MultiScribe Reverse Transcriptase, oligo(dT), and a random-hexamer primer mix (all from Applied Biosystems Life Technologies, CA, USA). Quantitative evaluation of the target genes was then performed using an ABI Prism 7500 sequence detection system (Applied Biosystems Life Technologies) with Power SYBR Green PCR Master Mix reagent and specific primers (hHO-1 [sense, 5′-gggtgatagaagaggccaagact-3′; antisense, 5′-agctcctgcaactcctcaaga-3′], hiNOS, also known as NOS2, [sense, 5′-cagcgggatgactttccaa-3′; antisense, 5′-aggcaagatttggacctgca-3′]). The PCR cycling conditions were 95 °C (15 s), 40 cycles at 95 °C (15 s), and 60 °C (1 min). According to melting point analysis, only one PCR product was amplified under these conditions. The relative quantity of a target gene, normalized to the β2-microglobulin gene as the endogenous control and relative to a calibrator, was expressed as 2^–ΔΔCt^ (fold difference).

### Transwell Migration Assay

Quiescent cells were seeded (1 × 10^5^ cells/well) into the upper chambers of 24-well Transwell inserts with polycarbonate membranes (Corning). The lower Boyden chambers received C1P, S1P, LPA, or LPC in 650 μL of assay medium, composed of RPMI medium with 0.5% BSA (Millipore; Billerica, MA, USA). The migrated cells were collected and scored using FACS analysis, and the results presented as a chemotactic ratio (the number of cells that migrated toward the medium containing C1P, S1P, LPA or LPC / the number of cells that migrated toward the assay medium alone × 100). In other experiments, quiescent KG1a hematopoietic cells were first incubated with SB203580 (20 μmol/L) for 6 h in serum-free medium. The cells were then washed and seeded onto the upper Transwell inserts, and their migration toward C1P, S1P, or SDF-1 was evaluated after a 3-h incubation. To analyze whether the increase in motility of malignant hematopoietic cells (U937) stimulated with bioactive lipids is a result of a directional (chemotactic) and/or a random (chemokinetic) response, we performed a checkerboard assay in which C1P (10 μM), S1P (0.01 *μ*M), LPA (10 *μ*M), or LPC (20 *μ*M) were added at the same time to the upper and the lower Transwell chambers.

### Adhesion of Malignant Hematopoietic Cells to Fibronectin

Cells were made quiescent for 5 h in 0.5% BSA RPMI 1640 medium in a humidified atmosphere of 5% CO_2_ at 37 °C. Next, the leukemia cells were cultured in the same medium in the presence or absence of C1P, S1P, LPA, or LPC. Cells (3 × 10^3^ cells/well) were then added directly and allowed to adhere to the fibronectin-coated wells in 96-well plates at 37 °C, as we previously outlined [41]. The results are presented as an adhesion ratio (the number of cells that adhered in the presence of the medium containing C1P, S1P, LPA, or LPC / the number of cells that adhered in presence of the assay medium alone × 100).

### Signal Transduction Studies

Quiescent cells were stimulated with 0.5% BSA in RPMI 1640 medium alone, C1P (10 μM for myeloid cells, 20 *μ*M for lymphoid cells), S1P (0.01 *μ*M for myeloid cells, 0.5 *μ*M for lymphoid cells), LPA (10 *μ*M for myeloid cells, 1 *μ*M for lymphoid cells), LPC (20 *μ*M for myeloid cells, 5 *μ*M for lymphoid cells), or SDF-1 (300 ng/mL) for 5 min at 37 °C. When required, the cells were also pretreated with SB203580 for 6 h in serum-free RPMI medium at 37 °C, and afterwards the cells were exposed to C1P, S1P, or SDF-1 at the indicated concentrations for 5 min at 37 °C. The protein lysates were extracted as described before [35] and then measured using the Pierce BCA Protein Assay kit (Pierce, Rockford, IL) and Multimode Analysis software (Beckman Coulter). The extracted, volume-adjusted protein (20 μg/each sample) was then separated on a 4–12% SDS-PAGE gel, and the fractionated protein was transferred to a PVDF membrane (Bio-Rad). All membranes were blocked with 2.5% BSA. Phosphorylation of the intracellular kinase p44/42 mitogen-activated protein kinase (p42/44 MAPK), p38 MAPK, and AKT was detected by incubating the membranes overnight at 4 °C with phosphospecific anti-p-p42/44 MAPK (clone no. 9101, diluted 1:1000) and anti-p-AKT (Ser473; clone no. 9271, diluted 1:1000) rabbit polyclonal antibodies and anti-p-p38 MAPK (Thr180/Thr182; clone no. 9216, diluted 1:2000) mouse monoclonal antibodies (Cell Signaling). Next, membranes were incubated with horseradish peroxidase (HRP)-conjugated goat anti-rabbit or anti-mouse IgG secondary antibodies (Santa Cruz Biotech, 1:5000). To confirm equal protein loading in all lanes, the blots were stripped using stripping buffer (Thermo Scientific) and then reprobed with appropriate anti-rabbit p42/44 MAPK (clone no. 9102), anti-rabbit p38 MAPK (clone no. 9212) or anti-rabbit AKT (clone no. 9272) antibodies (all from Cell Signaling). Enhanced chemiluminescence (ECL) reagent (Amersham Life Sciences) and film (Hyperfilm, Amersham Life Sciences) were used for band visualization using an automated film developer with fresh warm developer and fixer solutions.

### Detection of HO-1 by Western Blotting

Cells were cultured with C1P (10 μM), S1P (0.01 *μ*M), LPA (10 *μ*M), LPC (20 *μ*M), or SDF-1 (300 ng/mL) in serum-free RPMI 1640 medium for 6 h at 37 °C. KG1a hematopoietic cells were pretreated first with SB203580 for 6 h in serum-free medium, and after washing and replacement of the serum-free medium the cells were then exposed to C1P, S1P, or SDF-1 for a further 6 h at the indicated concentrations. The cells were harvested, centrifuged, and washed with ice-cold PBS for protein extraction (50 μg/each sample), and the membranes were blocked with 2.5% nonfat dry milk in Tris-buffered saline containing 0.1% Tween (TBST) for 1 h at room temperature. After washing with TBST, the membranes were incubated with rabbit anti-HO-1 polyclonal antibody (Enzo Life Sciences, NY, USA, diluted 1:1000) overnight at 4 °C. To assure equal protein loading in all lanes, the blots were then reprobed with rabbit anti-β-actin monoclonal antibody (Novus Biologicals, USA, diluted 1:1000).

### Cell Proliferation

Cells were cultured in 96-well plates (Cell Star; Greiner Bio-One) at an initial density of 3 × 10^4^ cells/mL in RPMI 1640 medium with 0.5% BSA in the presence or absence of bioactive lipids. The RPMI medium with 0.5% BSA was used as a negative control, while full medium containing 10% FBS was treated as a positive control. The cell number was calculated directly after cell seeding (0 h) as well as 24, 48, and 72 h after addition of stimulant. At the indicated time points, the cells were harvested from the wells and counted using FACS.

### Xenotransplantation into Immunodeficient Mice

The care and use of mice in this study was carried out in accordance with guidelines provided by the Institutional Animal Care and Use Committee of the University of Louisville that conforms to the Guide for Care and Use of Laboratory Animals (Department of Health and Human Services, publication no. ([NIH] 86–23). Prior to in vivo transplantation, KG1a cells (1 × 10^6^ per mouse) were treated ex vivo for 6 h with C1P (10 μM), S1P (0.01 *μ*M), or vehicle only as a control. In parallel, some cells were initially exposed to the small-molecule p38 MAPK inhibitor SB203580 (20 μmol/L) or vehicle alone for 6 h before bioactive lipids were added to the medium. All cells were then washed and transplanted into severe combined immunodeficient (SCID)-beige inbred mice (*n* = 3 per group), which were initially irradiated with 750 cGy at 24 h before transplantation. At 48 h post transplantation, bone marrows, livers, and lungs were collected, and the presence of metastasized cancer cells (i.e., murine–human chimeras) was evaluated as previously described [41].

### Chemotaxis and Clonogenic Assays

CD33^+^ blasts purified from primary human AML patients were also subjected to a transmigration assay in response to C1P and S1P in serum-free medium at the described concentrations and methods. At the same time, cell migration was also evaluated in response to medium containing SDF-1 or medium without stimulants. After a 3-h stimulation, the migrated cells were collected and plated with methylcellulose base medium, growth factors (GM-CSF [25 ng/mL] and IL-3 [10 ng/mL]), and after 14 days of culture at 37 °C and 5% CO_2_ atmosphere the numbers of CFU-GM colonies were scored using an inverted microscope (Olympus, Center Valley, PA, USA).

### Data Analysis

Statistical analysis was carried out using GraphPad Prism 6 (GraphPad Software Inc.) and Sigma software (Sigma Software Inc.). All data are presented as mean ± SD. Statistical analysis of the data was done using one-way ANOVA and Tukey’s test for post hoc pairwise multiple comparisons. In all analyses, *p* < 0.05, *p* < 0.01, and *p* < 0.001 were considered significant.

## Results

### Human Leukemia Cell Lines Express Receptors for Bioactive Phospholipids and Respond to Stimulation by Enhanced Motility and Adhesion but Not Proliferation

**Fig. 1:**
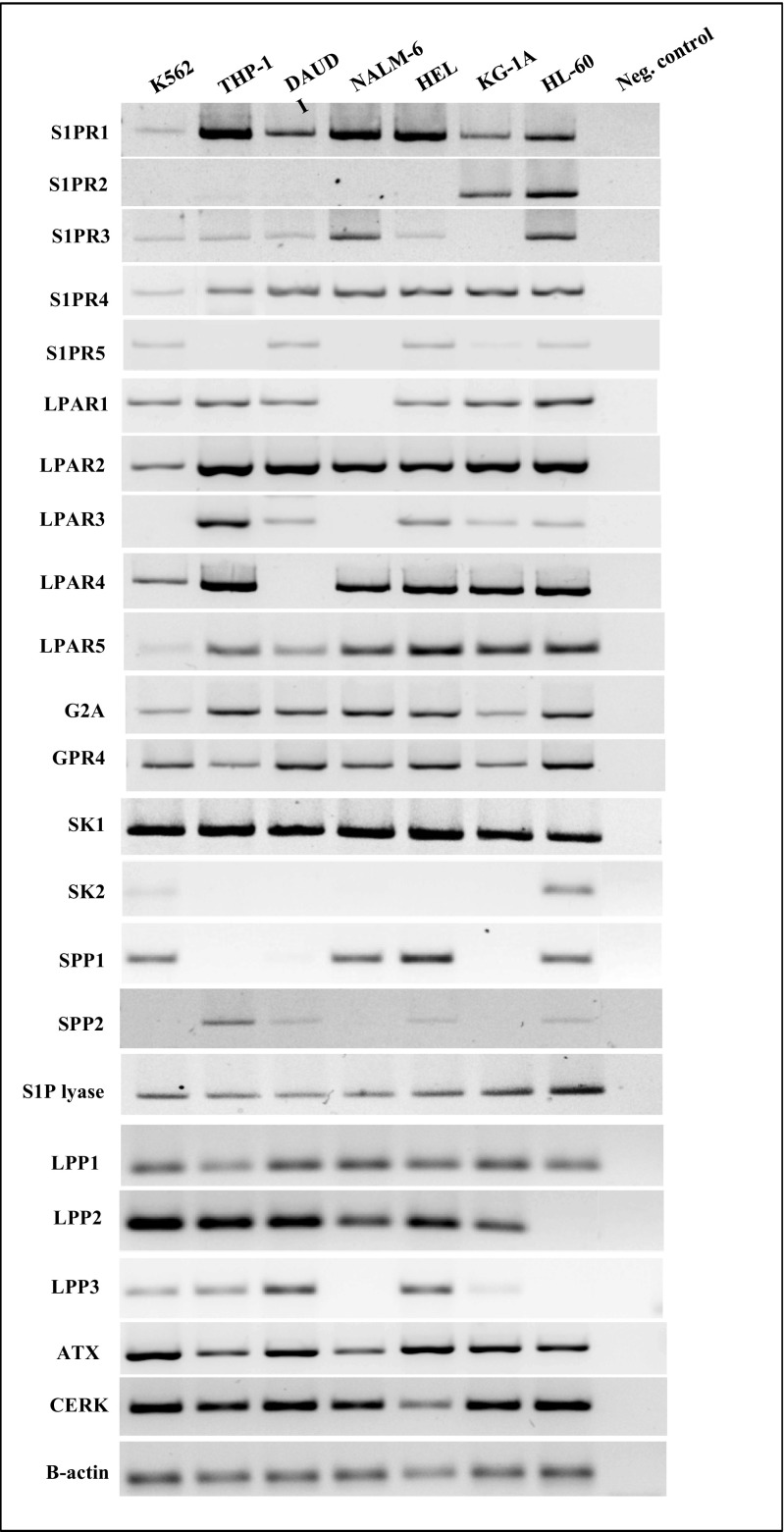
**Human malignant hematopoietic cell lines express functional S1P receptors (S1PR**_**1–5**_**), LPA receptors (LPAR**_**1–5**_**), LPC receptors (G2A and GPR4), and enzymes that are potentially involved in bioactive phosholipid biosynthesis and degradation.** Expression of these receptors was detected in purified mRNA samples from various myeloid and lymphoid leukemia cell lines by reverse transcription polymerase chain reaction (RT–PCR). Samples containing only water instead of cDNA and samples without reverse transcriptase were used in each run as negative controls. Representative agarose gels of the RT-PCR amplicons are shown

To demonstrate that the leukemic cells employed in our studies express receptors for S1P, LPA, and LPC, we evaluated the expression of these receptors at the mRNA level (Fig. 1) and found that several are expressed in human leukemic cell lines. However, we could not analyze the expression of receptor/s for C1P, as these receptors have not yet been cloned.

**Fig. 2:**
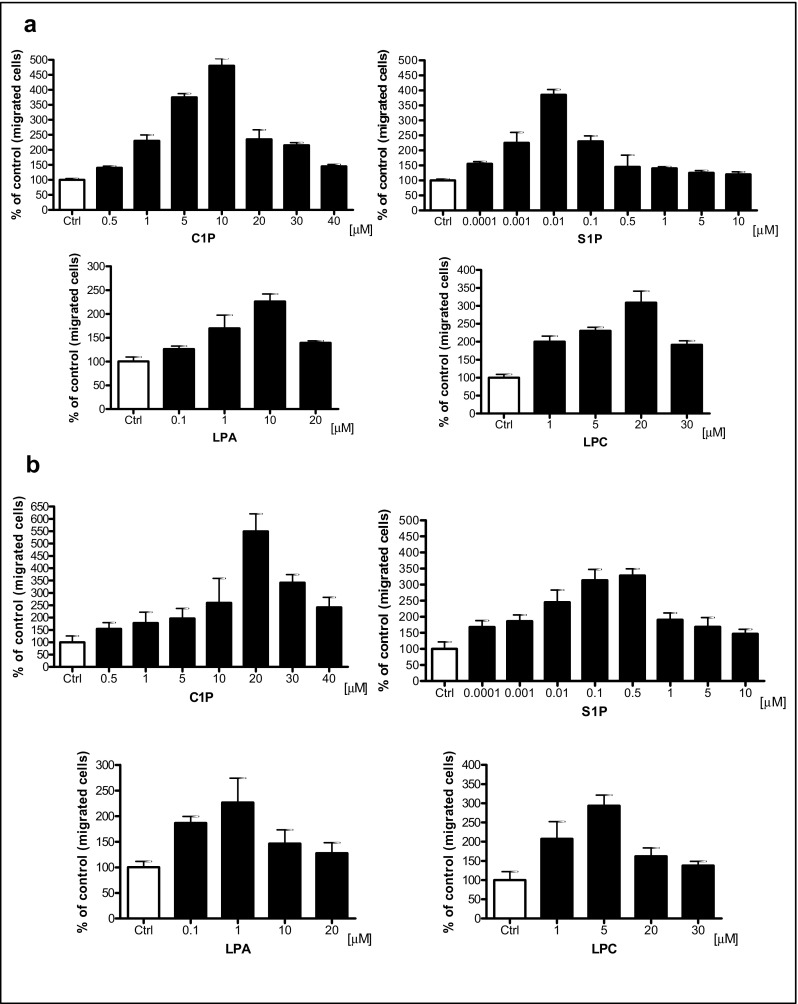

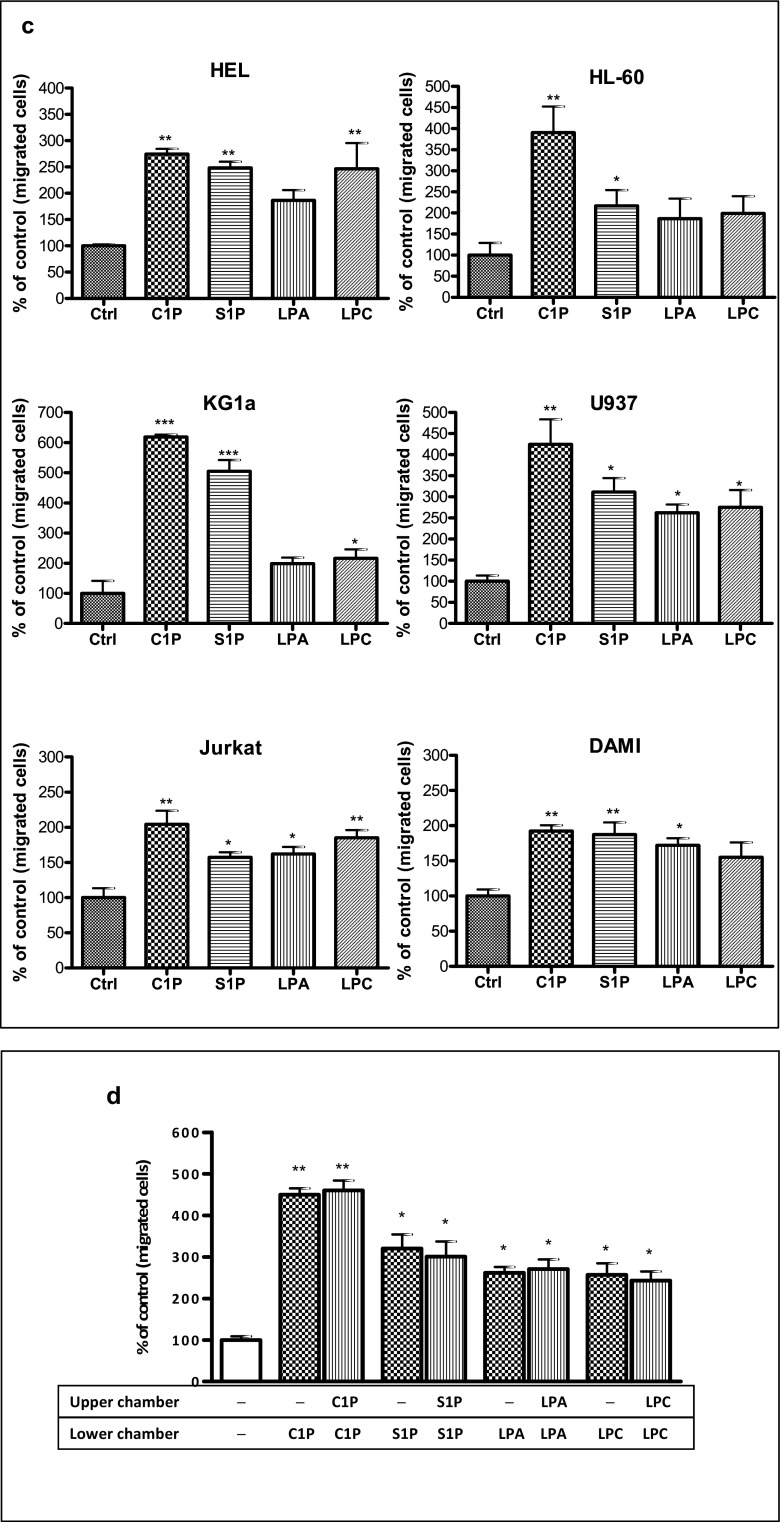
**Bioactive lipids enhance migration of human leukemia cell lines. Panel A.** Concentration-dependent effect of bioactive lipids on the chemotaxis of malignant myeloid cells (K562**). Panel B.** Concentration-dependent effect of bioactive lipids on chemotaxis of malignant lymphoid cells (Nalm6). **Panel C.** Chemotaxis of malignant myeloid and lymphoid cells through Transwell membranes (8-μm pore size) was assessed for fixed concentrations of C1P (10 μM for myeloid cells, 20 *μ*M for lymphoid cells), S1P (0.01 *μ*M for myeloid cells, 0.5 *μ*M for lymphoid cells), LPA (10 *μ*M for myeloid cells, 1 *μ*M for lymphoid cells), and LPC (20 *μ*M for myeloid cells, 5 *μ*M for lymphoid cells). Before stimulation, cells were rendered quiescent in serum-free medium for 5 h at 37 °C. All leukemia cell lines employed (10^5^ cells/100 μL/insert) were also evaluated for migration toward RPMI medium containing 0.5% BSA as a negative control. Three hours post-stimulation, the loaded inserts were carefully removed and the migrated cells harvested and counted by FACS analysis. Data are extracted from at least triplicate samples from three independent experiments. In all experiments, the negative control values are normalized to 100%. Data are displayed as means ± SD, with a statistical significance *p < 0.05, **p < 0.01, and ***p < 0.001 in migration assays between cells exposed to bioactive lipids versus unstimulated cells. **Panel D.** The increase in motility of malignant hematopoietic cells (U937) in response to bioactive lipids is the result of a random chemokinetic response. A checkerboard assay in which C1P (10 μM), S1P (0.01 *μ*M), LPA (10 *μ*M), or LPC (20 *μ*M) were added at the same time to the upper and the lower Transwell chambers. Data are displayed as means ± SD, with a statistical significance *p < 0.05 and **p < 0.01 versus unstimulated cells

Next, to address whether other phospholipids besides S1P, such as C1P, LPC, and LPA, enhance motility of human leukemic cells, we first performed Transwell migration dose-response experiments in the myeloid K-562 cell line and the lymphoid Nalm6 cell line with different doses of S1P, C1P, LPA, and LPC and observed that these cells responded in a dose-dependent manner (Fig. 2a, b). Based on these results, we employed optimal doses of these ligands in Transwell migration studies to evaluate the effect of bioactive phospholipids on the migration of HEL, HL-60, KG1a, U937, Jurkat, and DAMI cells (Fig. 2c). We observed that all these cell lines responded to bioactive phospholipids by enhanced motility.

Enhanced migration of cells in response to a potential chemoattractant could be the result of directed chemotaxis or random chemokinesis. To determine which one of these mechanisms is involved in migration of human leukemic cells, we performed a checkerboard Transwell migration assay in which a potential migration-inducing agent was added to the upper and lower chambers [2, 35]. As shown in the example of U937 cells, addition of bioactive phospholipids to the lower or upper chamber had the same effect on the number of cells accumulating in the lower Transwell chambers, which indicates a major involvement of chemokinesis (Fig. 2d).

**Fig. 3:**
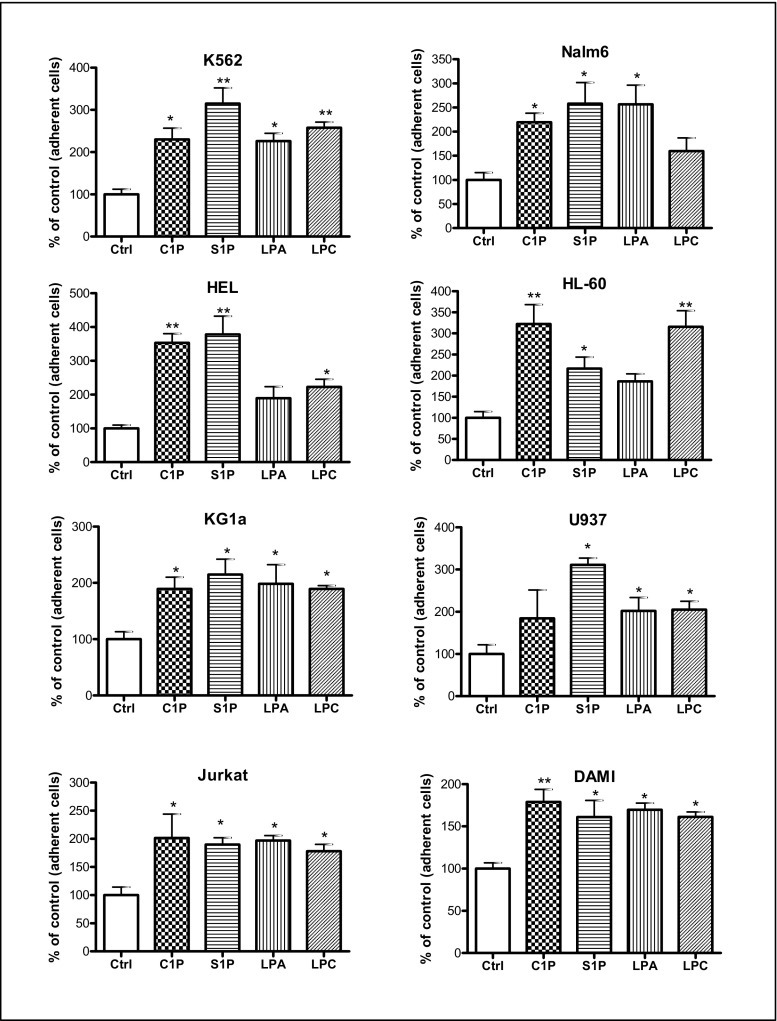
**Bioactive lipids increase the adhesiveness of human leukemia cell lines to fibronectin.** Adhesion of myeloid and lymphoid leukemia cell lines to fibronectin-coated surfaces was evaluated in response to C1P (10 μM for myeloid cells, 20 *μ*M for lymphoid cells), S1P (0.01 *μ*M for myeloid cells, 0.5 *μ*M for lymphoid cells), LPA (10 *μ*M for myeloid cells, 1 *μ*M for lymphoid cells), or LPC (20 *μ*M for myeloid cells, 5 *μ*M for lymphoid cells). Quiescent cells (3000 cells/100 μL) were stimulated in medium with 0.5% BSA for 5 min at 37 °C. After the non-adherent cells were removed via three consecutive washes with PBS, the number of adherent cells was directly scored by microscopic analysis. Cells were also evaluated for adhesion in RPMI medium containing 0.5% BSA as a negative control. Data are extracted from at least triplicate samples from three independent experiments. In all experiments, the negative control values are normalized to 100%. Data are displayed as means ± SD, with a statistical significance *p < 0.05 and **p < 0.01 in the adhesion assay between cells treated with bioactive lipids versus untreated cells

Next, we evaluated the effect of S1P, C1P, LPC, and LPA on the adhesion of established human leukemic cell lines (Fig. 3) and found that all of them enhanced the adhesion of human leukemic cells.

In parallel experiments, we also evaluated the effect of bioactive lipids on proliferation of leukemic cell lines, but to our surprise, we did not observe any proliferation-promoting effects (data not shown).

### Bioactive Phospholipids Induce p42/44 MAPK and AKT Signaling in Human Leukemic Cell Lines

**Fig. 4:**
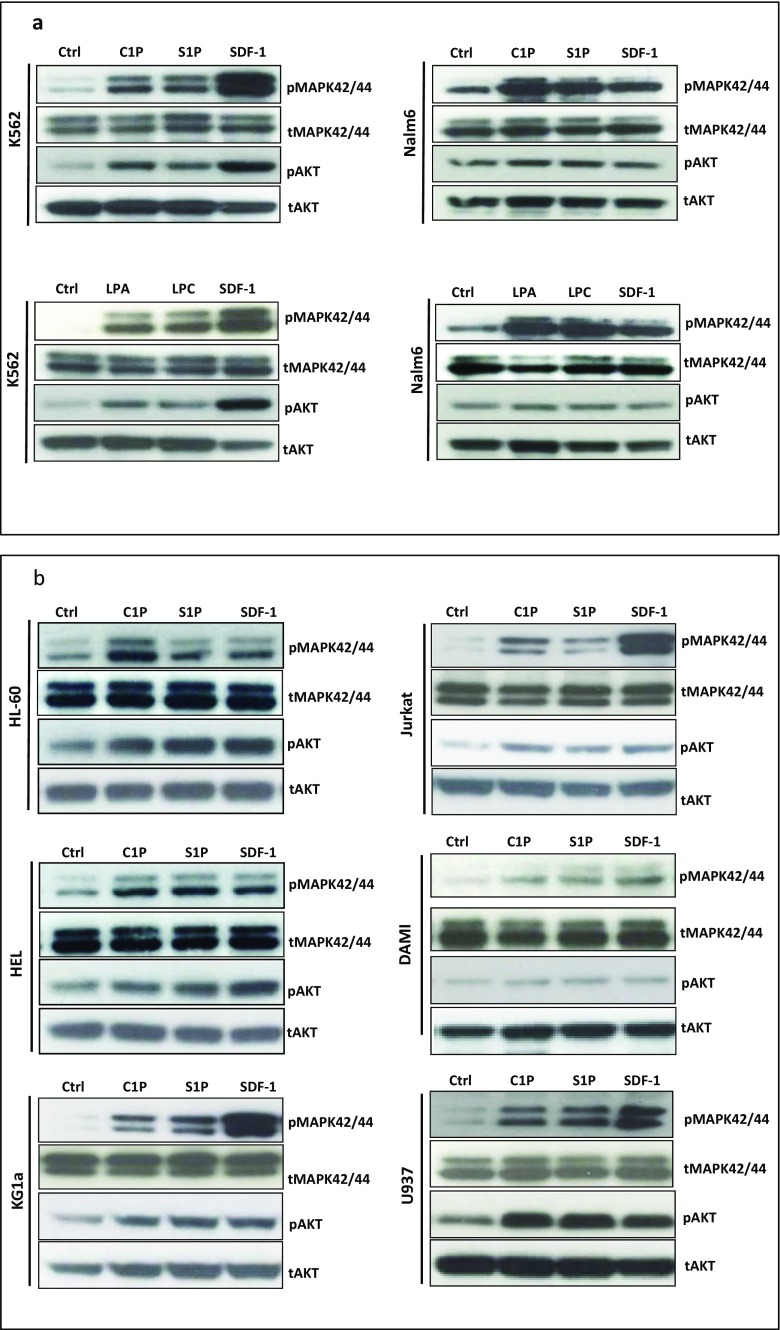
**Bioactive lipids activate the intracellular kinase p44/42 mitogen-activated protein kinase (p44/42 MAPK) and AKT in malignant human hematopoietic cell lines. Panels A and B.** The effect of bioactive lipids on phosphorylation of p42/44 MAPK and AKT^ser473^ intracellular pathway proteins in both myeloid and lymphoid leukemia cell lines was analyzed by western blotting. These cells (2 × 10^6^ cells/mL) were rendered quiescent for 5 h in RPMI medium containing 0.5% BSA at 37 °C, and afterwards the protein lysates were harvested after a 5-min stimulation with C1P (10 μM for myeloid cells, 20 μM for lymphoid cells), S1P (0.01 *μ*M for myeloid cells, 0.5 *μ*M for lymphoid cells), LPA (10 *μ*M for myeloid cells, 1 *μ*M for lymphoid cells), or LPC (20 *μ*M for myeloid cells, 5 *μ*M for lymphoid cells). Cells were also treated with SDF-1 only (300 ng/mL) or with serum-free medium. The experiment was carried out twice with similar results, and representative blots are shown

To obtain additional proof that human leukemic cell lines express functional receptors for the bioactive phospholipids tested in our studies, we performed signal transduction experiments and observed that stimulation of all our cell lines by S1P and C1P induced phosphorylation of p42/44 MAPK (Fig. 4) and, except in Nalm6 cells, phosphorylation of AKT. We also found that stimulation of K562 and Nalm6 cells by LPA and LPC similarly led to phosphorylation of p42 MAPK and AKT (Fig. 4a). Moreover, all cell lines employed in our studies also responded robustly to stimulation by SDF-1 (Fig. 4), as expected.

### Bioactive Phospholipids Downregulate Expression of HO-1 and iNOS in a p38 MAPK-Dependent Manner

**Fig. 5:**
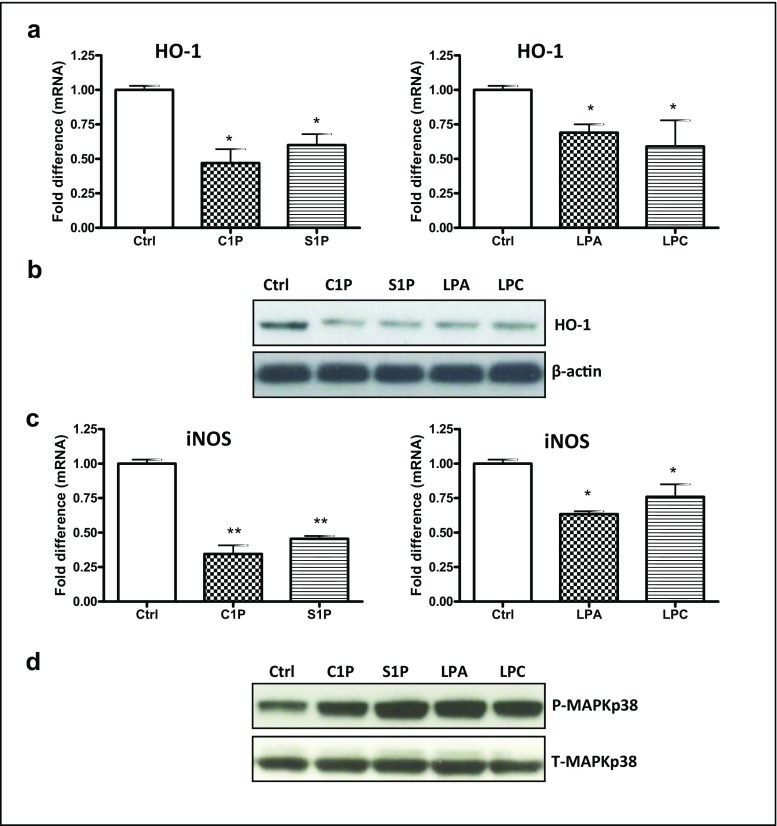
**Bioactive lipids enhance the migration of leukemic cells through phosphorylation of p38 MAPK-dependent downregulation of heme oxygenase 1 (HO-1) and inducible nitric oxide synthase (iNOS). Panels A and C.** RT-qPCR analysis of mRNA transcripts for human HO-1 (**Panel A**) and iNOS (**Panel C**) in mRNA samples purified from KG-1a cells incubated with C1P (10 *μ*M), S1P (0.01 *μ*M), LPA (10 *μ*M), or LPC (20 *μ*M) in serum-free medium for 6 h at 37 °C. β2-microglobulin was used as an endogenous control. Samples containing water only instead of cDNA were used in each run as a negative control. *p < 0.05 and **p < 0.01 are considered statistically significant between cells exposed to bioactive lipids versus unstimulated cells. **Panel B.** Western blot for human HO-1 in protein lysates collected from KG-1a cells (20 μg per sample). After incubation of cells with C1P, S1P, LPA, or LPC at the doses indicated above, the protein was immediately extracted and afterwards quantified using the Pierce BCA Protein Assay kit and Multimode Analysis software. In parallel, β-actin was also analyzed to ensure the equivalence of loading. Proteins extracted from cells cultured in assay medium only served as a control. **Panel D.** Western blot analysis for phospho-p38 MAPK in protein lysates collected from quiescent KG-1a cells. Cells were stimulated with 0.5% BSA in RPMI 1640 medium, C1P (10 *μ*M), S1P (0.01 *μ*M), LPA (10 *μ*M), or LPC (20 *μ*M) for 5 min at 37 °C. Total p38 MAPK was also analyzed to ensure equal protein loading in all lanes

In our previous studies we reported that HO-1 and iNOS are potent inhibitors of hematopoietic cell migration [2, 35–39]. We also reported that this effect is mediated by upregulation of p38 MAPK [35]. Here we tested the effect of S1P, C1P, LPC, and LPA on expression of HO-1 (Fig. 5a, b) and iNOS (Fig. 5c) and observed that bioactive phospholipids downregulate expression of these inflammation-controlling enzymes. Moreover, this effect was mediated by upregulation of p38 MAPK, as shown in Fig. 5d.

**Fig. 6:**
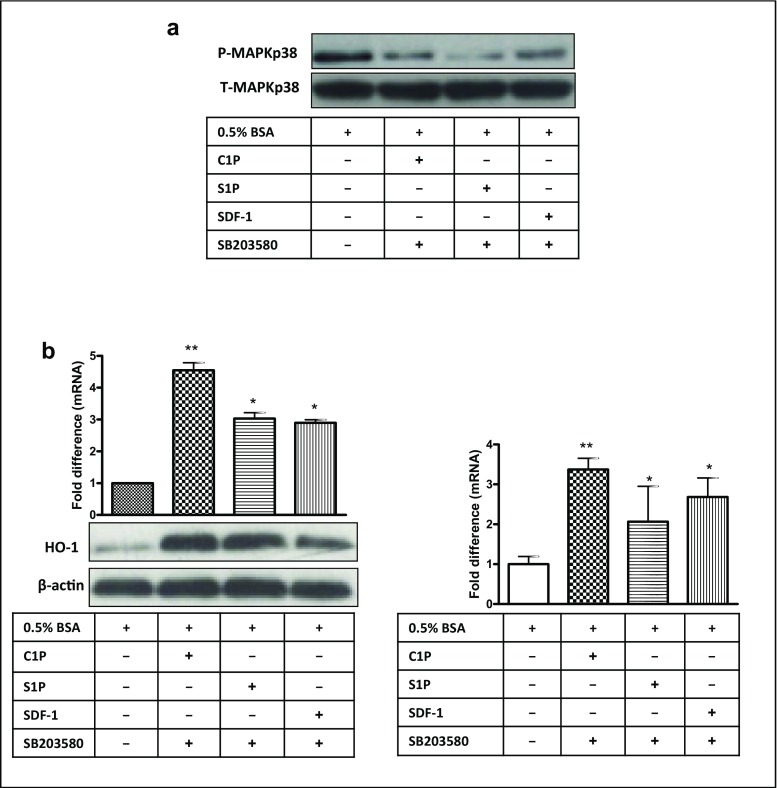
**Inhibition of p38 MAPK impedes migration of leukemic cells by upregulation of HO-1 and iNOS. Panel A.** Western blot analysis for phospho-p38 MAPK in protein lysates collected from quiescent KG-1a cells. Cells were pretreated with SB203580 (20 μmol/L) for 6 h at 37 °C in serum-free medium. Cells were afterwards washed with PBS and stimulated with 0.5% BSA in RPMI 1640 medium, C1P, S1P, or SDF-1 for 5 min at 37 °C. Total p38 MAPK was also analyzed to confirm equal loading of protein in all lanes. **Panel B.** In parallel, HO-1 (left) and iNOS (right) expression was evaluated at both the mRNA (RT-qPCR) and protein (western blotting) levels in KG-1a cells (bottom, left). The mRNA samples and protein lysates were obtained after their pretreatment with SB203580 for 6 h, followed by washing with PBS and a further 6-h exposure in serum-free medium at 37 °C to C1P, S1P, or SDF-1. In the two latter experiments, β2-microglobulin and β-actin were employed as endogenous controls, respectively. In the RT-qPCR assays, samples containing only water instead of cDNA were used in each run as a negative control. Cells kept unstimulated (with serum-free medium only) served as a negative control. *p < 0.05 and **p < 0.01 are considered significant versus the unstimulated cells

Next, we stimulated KG-1a cells with C1P, S1P, or SDF-1 and evaluated their effect on expression of p38 MAPK in the presence of the specific inhibitor SB203580 and confirmed that p38 MAPK phosphorylation was inhibited in a SB203580-dependent manner (Fig. 6a). In agreement with this observation, subsequent exposure of KG-1a cells to SB203580 upregulated expression of HO-1 and iNOS, both at the protein and mRNA levels (Fig. 6b, left and right panels, respectively).

### Bioactive Phospholipids Enhance the Spread of Intravenously Injected KG-1a Cells in Immunodeficient Mice, and Inhibition of p38 MAPK Attenuates this Effect

**Fig. 7:**
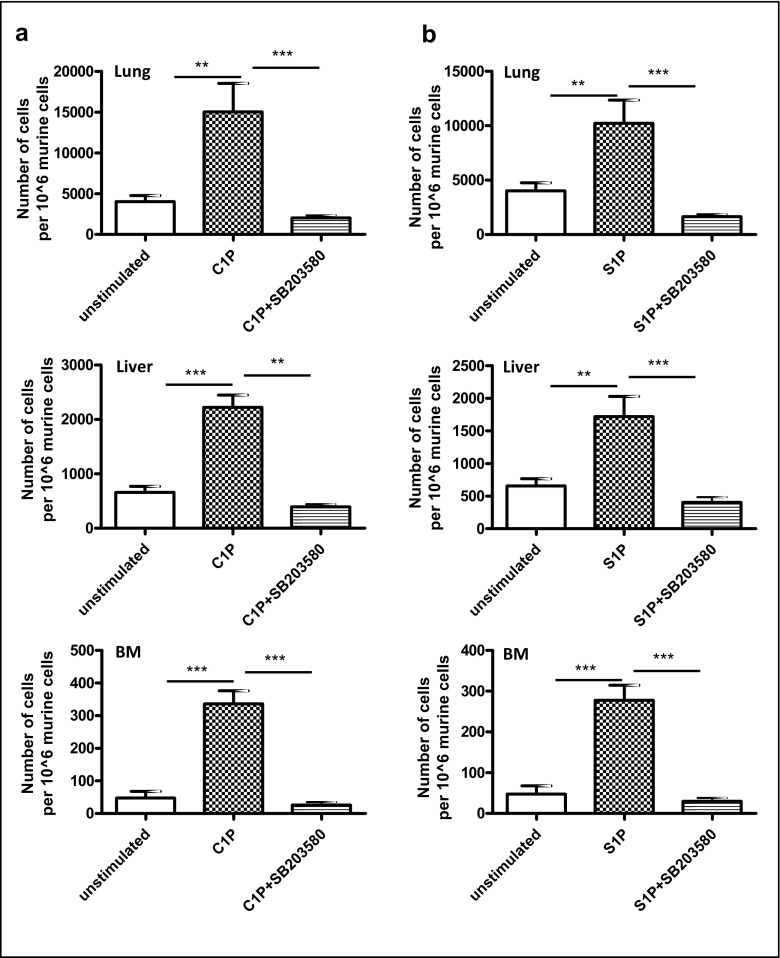
**Bioactive lipids enhance metastasis*,*whereas inhibition of p38 MAPK hinders the metastasis of leukemic cells in vivo*.*Panels A and B.** Detection of transplanted human KG-1a cells (1 × 10^6^ per mouse) in the organs of irradiated (SCID)-beige inbred mice post in vivo transplantation. Pre-implantation, the cells were incubated ex vivo with vehicle only, C1P (10 *μ*M) only, or C1P (10 *μ*M) after incubation of cells with SB203580 (**Panel A**) and S1P (0.01 *μ*M) only, or S1P (0.01 *μ*M) after incubation of cells with SB203580 (**Panel B**) for 6 h. In all conditions, serum-free medium was used. Detection of human cells in lungs (**top row**), livers (**middle row**), and BM (**bottom row**) was evaluated 48 h after cell transplantation into irradiated SCID mice by RT-qPCR for the presence of human Alu sequences in purified genomic DNA samples. For statistical comparisons, a one-way analysis of variance and a Tukey’s test for post hoc analysis were carried out, and means ± SD are shown. Significance levels are indicated at **p < 0.01, and ***p < 0.001 versus untreated cells

To test whether bioactive phospholipids enhance the metastatic spread of human leukemic cells, KG-1a cells were exposed to C1P (Fig. 7a) or S1P (Fig. 7b) before injection into immunodeficient mice. We observed that increased numbers of KG-1a cells that were pre-stimulated before iv. injection into mice with C1P or S1P were seeded into lungs, liver and bone marrow. This effect was attenuated in these cells after downregulation of p38 MAPK by exposure to SB203580. Based on this finding, we conclude that inhibition of p38 MAPK attenuates the spread of leukemic cells exposed to bioactive phospholipids.

### C1P and S1P Enhance the Migration of Leukemic Blasts

**Fig. 8:**
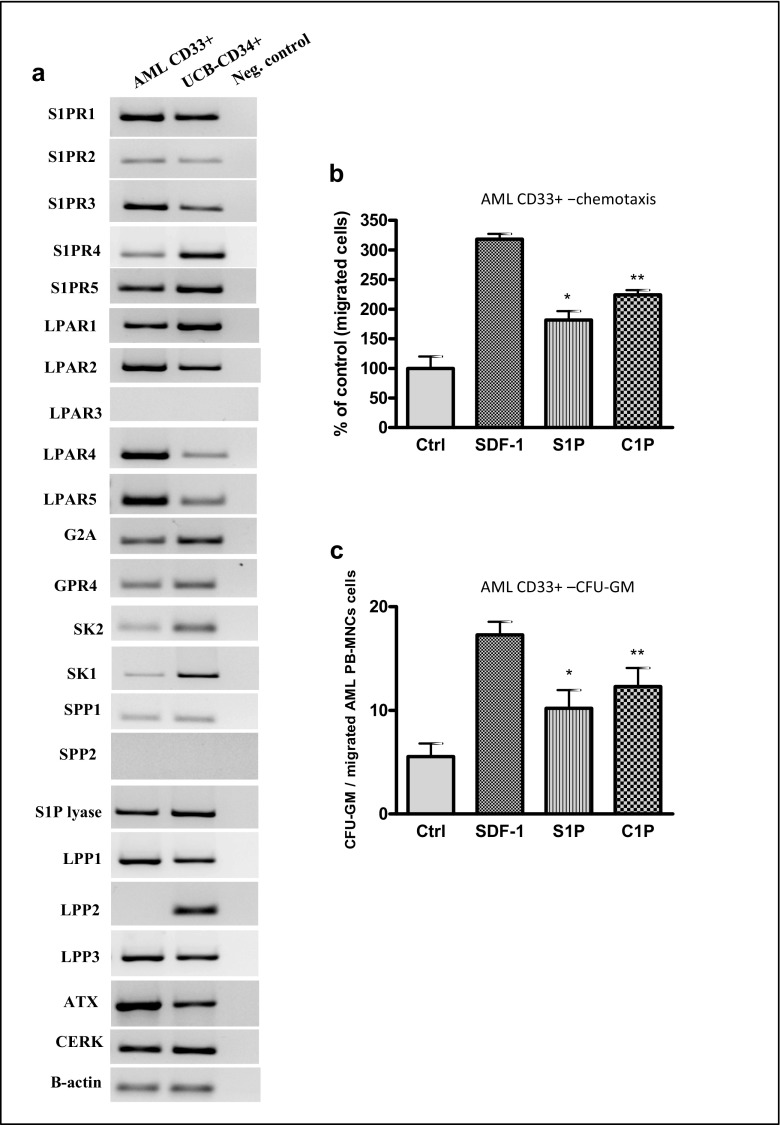
**Bioactive lipids accelerate migration of primary human CD33**^**+**^**AML cells. Panel A.** Human CD33^+^ AML cells and normal CD34^+^ cells express functional S1P receptors (S1PR_1–5_), LPA receptors (LPAR_1–5_), LPC receptors (G2A and GPR4), and enzymes involved in bioactive phosholipid metabolism. **Panel B.** Transmigration of primary CD33^+^ AML blasts through Transwell membranes in response to C1P or S1P at the indicated concentrations was analyzed. AML-derived CD33^+^ cells were resuspended in RPMI 1640 medium with 0.5% BSA. SDF-1 was used as a positive control, whereas serum-free medium was used as a negative control. **Panel C.** Next, the migrated AML CD33^+^ cells were harvested and counted by FACS analysis and a CFU-GM clonogenic assay was immediately performed on the migrated cells. Data are displayed as means ± SD, with a statistical significance compared with the control, *p < 0.05 and **p < 0.01

Finally, we studied the effect of bioactive phospholipids on the migration of human CD33^+^ blasts isolated from AML patients (Fig. 8). We observed that both AML blasts and normal umbilical cord blood CD34^+^ cells express several receptors for phospholipids and the enzymes involved in their metabolism (Fig. 8a). Moreover, C1P and S1P enhanced migration of CD33^+^ blasts (Fig. 8b), and the CD33^+^ blasts were clonogenic for CFU-GM cells (Fig. 8c). Interestingly, in contrast to established leukemic cell lines (Fig. 2), neither LPC nor LPA was able to induce motility of blasts isolated from primary patients (data not shown).

## Discussion

The most important finding of this study is that human leukemic cells as well as patient AML blasts express a broad panel of functional receptors for signaling phospholipids and respond to their stimulation by downregulating synthesis of HO-1 and iNOS.

The overall rationale for performing this study was that, in contrast to S1P, the effects of C1P, LPC, and LPA on leukemic cells are not well known. Specifically, while S1P has been reported to be involved in the pathogenesis of CML, AML, ALL, and multiple myeloma and to chemoattract leukemic cell lines [12–15], there is no data on the role of C1P. Similarly, there was very limited information about the effect of LPC and LPA on leukemic cells.

Of the two phosphosphingolipids, S1P and C1P, a significant number of studies on hematopoietic cells have been performed with S1P, which is produced by phosphorylation of sphingosine by two sphingosine kinases (Sphk1 and Sphk2). Knockout of both kinases is lethal, indicating the important role of S1P in development. S1P is transported in the blood by erythrocytes, albumin, and high density lipoprotein (HDL) and degraded by S1P lyase. S1P activates five G protein-coupled receptors (S1PR_1–5_) and is a known regulator of vascular and immune systems, being an important regulator of the trafficking of T and B cells. S1P also plays a crucial role in egress of hematopoietic stem progenitor cells (HSPCs) from bone marrow into peripheral blood and is involved in the homing of these cells after transplantation [4, 17, 42].

In our experiments, the mRNAs for all S1P receptors were expressed by normal hematopoietic cells and CD33^+^ AML blasts. We also observed that human leukemic cell lines express functional S1P receptors, and S1PR_1_ and S1PR_4_ were expressed by all cell lines. Interestingly, the S1PR_1_ receptor is subject to inhibition by small-molecule inhibitors, and it has been proposed that this receptor mediates anti-apoptotic and pro-proliferative responses in human AML cells by inhibiting the generation of mitochondria-associated reactive oxygen species (ROS) [15]. These earlier studies were performed with HL60, U937, and K562 cell lines in which S1PR_1_ was overexpressed or knocked down [15]. In contrast to these earlier results, here we did not observe an effect of S1P on proliferation of established leukemic cell lines and primary patient leukemic cells, and the observed biological effects were mainly related to promoting cell trafficking and adhesion.

The effect of bioactive phospholipids on cell migration was based mainly on chemokinesis and not on classical chemotactic effects. Although increased random chemokinetic motility of leukemic cells also plays a role in the spread of leukemic cells throughout the body, the chemokinetic effect can be skewed toward enhanced chemotaxis in the presence of other chemotactic factors, such as SDF-1, as we demonstrated in the past [1, 2, 8, 35].

In contrast to S1P, the other bioactive phosphosphingolipid, C1P, is still understudied. C1P and its precursor ceramide have opposite effects on cell survival, and a correct balance between the concentrations of C1P and ceramide is essential for cell and tissue homeostasis. In contrast to S1P, C1P is most likely not secreted by intact cells but is released by leaky or damaged cells [43]. Although some authors claim that it stimulates cell proliferation, we did not observe such an effect in the leukemic cells evaluated in our studies. C1P was initially reported to stimulate migration of murine RAW264.7 macrophages [16]. In our past studies we reported that it is an important factor mediating migration of normal HSPCs, and here we show its stimulatory effect on migration in several human leukemic cell lines. Since the receptors for C1P have not been identified, we were not able to study their expression. However, our cell signaling experiments revealed that cells responded robustly to stimulation by C1P with phosphorylation of p42/44 MAPK and AKT.

Two other phospholipids studied in our work, LPC and LPA, are closely related, as LPC is rapidly converted in biological fluids to LPA by the enzyme autotaxin, which has lysophospholipase D activity. LPC has been described in the literature as recruiting phagocytic cells when released from the cells [44, 45]. In our experiments we demonstrated for the first time that LPC induces chemokinesis in human leukemic cells. Recently, an orphan G protein-coupled receptor, G2A, was identified that acts as a high-affinity receptor for LPC and is expressed mostly in lymphocytes [27]. LPC may also bind with low affinity to another G protein-coupled receptor, GPR4, which is also sensitive to lysophospholipids and protons [28]. We found that normal and malignant cells employed in our studies expressed both receptors. In one of these studies, LPC was reported to be a chemoattractant for DO11.10 T cell hybridoma and human AML cells [46, 47]. This latter effect seems to be indirect and mainly attributable to LPA generated enzymatically from LPC by autotaxin in an autocrine/paracrine manner [47]. Thus, the major biological role of LPC is as a substrate for synthesis of LPA in an autotaxin-dependent manner. We cannot rule out this effect in our study, as all of the cell lines evaluated in our work express autotaxin.

LPA acts in some cells as a potent mitogen due to its activation of five high-affinity G protein-coupled receptors, LPAR_1–5_, and activates the small GTPase Rho and Rho kinase. This can lead to the formation of stress fibers and enhanced cell migration through the inhibition of myosin light-chain phosphatase. LPA has also been demonstrated to stimulate proliferation of leukemic cells. However, we did not observe this effect in our studies. To explain this lack of an effect, it should be noted that several biological effects of LPA are mediated by the release of certain mitogens from LPA-stimulated cells. This effect of LPA on B cell malignancies and CML was reported to be indirect and related to induction of VEGF expression [48–51]. However, in agreement with other reports, we demonstrate that LPA induces migration of leukemic cells due to the induction of chemokinesis.

What is also important plasma level of bioactive phospholipids follows circadian rhythm of changes [52]. Moreover, they promote migration of very small embryonic like stem cells [53, 54] that may become specified into hematopoietic stem cells [55]. The potential transformation of these cells into leukemic cells requires further studies [55, 56].

Migration of cells is positively regulated by several peptide and non-peptide factors. As we recently observed, HO-1 and iNOS induced in normal and leukemic hematopoietic cells have a negative effect on cell trafficking. In agreement with these reports, we observed here that bioactive lipids stimulate migration of leukemic cells by downregulating expression of HO-1 and iNOS in a p38 MAPK-dependent manner. These results indicate that small-molecule inhibitors of p38 MAPK or small-molecule stimulators of both enzymes could find practical application as drugs by decreasing the spread of leukemic cells in the body and as supportive agents in anti-leukemic therapies.

In conclusion, human leukemic cells express a broad repertoire of functional receptors for bioactive phospholipids, and S1P, C1P, LPC, and LPA tested in our experiments increase the motility of these cells. Based on these results and taking into consideration the redundancy of this system, instead of inhibiting selected ligand–receptor axes, a more optimal strategy would be inhibiting the motility of leukemic cells by targeting common downstream events. For example, targeting p38 MAPK expression or upregulating HO-1 or iNOS seems to a better strategy to ameliorate the effects of phospholipids on leukemic cells.
